# Prognostic Metabolite Biomarkers for Soft Tissue Sarcomas Discovered by Mass Spectrometry Imaging

**DOI:** 10.1007/s13361-016-1544-4

**Published:** 2016-11-21

**Authors:** Sha Lou, Benjamin Balluff, Arjen H. G. Cleven, Judith V. M. G. Bovée, Liam A. McDonnell

**Affiliations:** 1 Center for Proteomics and Metabolomics, Leiden University Medical Center, Leiden, The Netherlands; 2 Maastricht MultiModal Molecular Imaging institute (M4I), Maastricht University, Maastricht, The Netherlands; 3 Department of Pathology, Leiden University Medical Center, Leiden, The Netherlands; 4 Fondazione Pisana per la Scienza ONLUS, Pisa, Italy

**Keywords:** Metabolites, Prognosis, Biomarker discovery, MALDI-MSI, High grade sarcoma, Leiomyosarcoma, Myxofibrosarcoma, Osteosarcoma, Undifferentiated pleomorphic sarcoma, Soft tissue sarcoma

## Abstract

Metabolites can be an important read-out of disease. The identification and validation of biomarkers in the cancer metabolome that can stratify high-risk patients is one of the main current research aspects. Mass spectrometry has become the technique of choice for metabolomics studies, and mass spectrometry imaging (MSI) enables their visualization in patient tissues. In this study, we used MSI to identify prognostic metabolite biomarkers in high grade sarcomas; 33 high grade sarcoma patients, comprising osteosarcoma, leiomyosarcoma, myxofibrosarcoma, and undifferentiated pleomorphic sarcoma were analyzed. Metabolite MSI data were obtained from sections of fresh frozen tissue specimens with matrix-assisted laser/desorption ionization (MALDI) MSI in negative polarity using 9-aminoarcridine as matrix. Subsequent annotation of tumor regions by expert pathologists resulted in tumor-specific metabolite signatures, which were then tested for association with patient survival. Metabolite signals with significant clinical value were further validated and identified by high mass resolution Fourier transform ion cyclotron resonance (FTICR) MSI. Three metabolite signals were found to correlate with overall survival (*m/z* 180.9436 and 241.0118) and metastasis-free survival (*m/z* 160.8417). FTICR-MSI identified *m/z* 241.0118 as inositol cyclic phosphate and *m/z* 160.8417 as carnitine.

**Electronic supplementary material:**

The online version of this article (doi:10.1007/s13361-016-1544-4) contains supplementary material, which is available to authorized users.

**Graphical Abstract:**
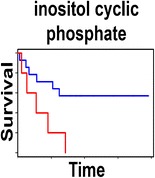
ᅟ

## Introduction

Soft tissue sarcomas are a rare group of tumors, comprising less than 1% of all malignant tumors [1] with more than 50 histologic subtypes [2]. About 10% of patients with soft tissue sarcoma have detectable metastases (most common in the lungs) at diagnosis of the primary tumor, and at least one-third of patients die from tumor-related disease (most of them from lung metastases) [1]. Additionally, different tumor subpopulations can have different clinical-pathological behavior [3–5].

The combination of rarity, many subtypes with overlapping and heterogeneous histologies, and molecular intratumor heterogeneity have made the correct diagnosis and treatment of high-grade sarcomas challenging. Different therapeutic strategies have been developed but the choice is dependent on subtype because they confer different chemosensitivity [6]. Accurate histologic classification of sarcomas can be far from straightforward: full concordance between primary diagnosis and second opinion was observed in less than 60% [7]. Additionally, prognostic factors associated with local failure and overall survival are also dependent on tumor location and size [1]. There is an urgent need for more objective molecular predictors to improve prognosis and treatment.

A cell’s metabolome has been defined as the unique chemical fingerprints left behind by a cell’s specific molecular process [8], and altered metabolism is now one of the hallmarks of cancer [9]. Cancer cells can exhibit significant alterations in metabolic pathways such as glycolysis, respiration, the tricarboxylic acid cycle, oxidative phosphorylation, lipid metabolism, and amino acid metabolism [10]. Tumors show various metabolic aberrations but perhaps the most central to tumor proliferation is the ‘Warburg effect’ [11]. Cancer cells reprogram their energy metabolism by limiting energy metabolism to glycolysis, producing excessive levels of lactate, even under normoxic conditions (“aerobic glycolysis”). The high rate of glucose uptake and glycolysis in tumors is the basis for the widely used tumor imaging technique [18F] deoxyglucose-positron emission tomography (18FDG PET) [12–14].

The molecular basis for increased glycolysis and altered metabolism is currently an active area of onco-metabolomic research, and spans the identification and validation of biomarkers in the cancer metabolome that can stratify high-risk patients and/or distinguish between benign and advanced metastatic forms of the disease to the functional consequences of the altered metabolic states [15, 16].

Previous studies on sarcoma-metabolomics has mostly concerned cell lines [17–19] because of the much greater ease of freezing their metabolome status; the metabolome reacts quickly to changes in oxygen, blood supply, etc. Nevertheless, it is important to assess metabolites within the histopathologic context of patient tumor samples, especially so for histologically heterogeneous tumors such as sarcomas.

Mass spectrometry imaging (MSI) has had a rapid and substantial impact on clinical research [20] because of its ability to directly visualize a tissue’s molecular content without any labeling or a-priori knowledge [21–23]. MSI enables, through a seamless integration of histology with the MSI data, cell-type specific molecular signatures to be obtained from the real histopathologic context of patient tissues [24, 25]. Both matrix assisted laser desorption/ionization (MALDI) and desorption electrospray ionization (DESI) have been used to acquire metabolite MSI datasets of cancer tissues. For example MALDI MSI may be used to visualize metabolites and metabolic pathways in fresh frozen tumor tissues [26] and identify biomarkers, including in formalin fixed paraffin embedded tissues and tissue microarrays [27]; DESI has been used to identify metabolite biomarkers for discriminating between different brain and breast tumors [28, 29]. Combining tumor-specific metabolite signatures with patient follow-up data has revealed prognostic biomarkers [27]. Though less established for clinical research than protein MSI, these studies indicate that the metabolite signatures obtained via MSI can also be used to differentiate between different tumor entities and between patient groups.

MSI has previously been applied in sarcomas. Caldwell et al. used MALDI MSI to investigate protein changes in the tumor microenvironment of malignant fibrous histiocytoma [30]. MALDI MSI of proteins has revealed molecular intra-tumor heterogeneity in myxofibrosarcoma [5], and it has been used to determine proteins and lipids that are tumor type- and tumor grade-specific for myxoid liposarcoma and myxofibrosarcoma [4]. However, an assessment of the prognostic value of metabolites for soft tissue sarcomas has not been reported to date. In this study, we investigated if the metabolite and lipid signatures detected by MALDI MSI using 9-aminoacridine in high-grade sarcomas, including high-grade leiomyosarcoma, myxofibrosarcoma, undifferentiated pleomorphic sarcoma, and osteosarcoma, can be associated with patient prognosis.

## Experimental

### Tissue Specimens and Sample Cohorts

Fresh frozen tumor samples of high-grade myxofibrosarcoma (MFS), leiomyosarcoma (LMS), undifferentiated pleomorphic sarcoma (UPS), and osteosarcoma (OS) were collected and handled as described previously [3]. Briefly, tissue samples were obtained from the archive of the Department of Pathology of Leiden University Medical Center (LUMC), The Netherlands. All tumor samples were acquired during routine patient care and were handled in a coded manner according to the ethical guidelines described in “Code for Proper Secondary Use of Human Tissue in The Netherlands” of the Dutch Federation of Medical Scientific Societies. Slides were reevaluated histologically and classified according to the 2013 World Health Organization criteria. MFS and LMS cases were histologically graded according to the French Fédération Nationale des Centres de Lutte Contre le Cancer. The final cohort comprised 8 LMS, 10 MFS, 8 UPS, and 7 OS patients (n_total_ = 33). Table 1 shows the clinic-pathological data of the patient series.

**Table 1:** Clinic-Pathological Characteristics of the Patient Series

		High grade leiomyosarcoma	High grade myxofibrosarcoma	High grade undifferentiated pleomorphic sarcoma	High grade osteosarcoma
No. of patients		8	10	8	7
Gender	Male versus female	4 versus 4	3 versus 7	6 versus 2	4 versus 3
Age	Median (y)	60.1	74.0	69.5	21.0
Therapy	Neoadjuvant treatment	1 / 8	0 / 10	1 / 8	5/ 7
	Adjuvant treatment	7 / 8	6 / 10	5 / 8	6 / 7
Length of follow-up	Median overall survival (mo)	25.3	64% survival probability at max. follow-up time (103.2 mo)	21.5	28.5
	Median metastasis-free survival (mo)	10.2	73% metastasis-free probability at max. follow-up time (52.0 mo)	11.1	9.5

### Tissue Preparation

A semi-supervised block randomization was used to distribute the patient tissue sections between and within slides in order to minimize any potential sources of bias during MSI data acquisition (pseudo-code available in Carreira et al. [31]).

Twelve micron-thick sections were cut at –20 °C in a cryostat and thaw-mounted onto indium-tin-oxide glass slides (Bruker Daltonik, Bremen, Germany) that had previously been coated with poly-L-lysine [32]. The mounted tissue sections were stored at –80 °C until use. Slides were first dried in a freeze dryer for 15 min, then fiducial markers added near the tissues using a Tipp-Ex pen. Tissue sections were sprayed with 10 mg/mL 9-aminoacridine in 70% methanol using the ImagePrep spraying device (Bruker Daltonik). The spraying method consisted of 22 cycles of 0.1 V for matrix layer thickness at 20% power and 10% modulation with 1.5 s for nebulization, 10 s of incubation, and 30 s of drying. Prior to the MSI data acquisition the matrix-coated slides were placed in the MSI slide holder and scanned with 2400 dpi resolution (Epson V200 Photo). Note the resolution of the scanned image was much higher than that of the MSI data (100 μm pixel size corresponds to 254 dpi) to enable accurate co-registration with the histological image.

### MSI Data Acquisition

All MSI experiments were performed using an Ultraflextreme III MALDI-ToF/ToF mass spectrometer (Bruker Daltonik) with a lateral resolution of 100 μm, a laser focus setting of “large” (corresponding to an on-target laser spot size of approximately 80 μm), and 500 accumulated laser shots per pixel (10 laser shots per step of a random walk within each pixel). Negatively charged ions up to *m/z* 1000 were detected with a digitization rate of 4 GHz in reflectron mode. All pixel mass spectra were processed with a smoothing algorithm (Savitzky-Golay algorithm, width 0.005 *m/z*, two cycles) and a background subtraction (TopHat algorithm) during data acquisition using FlexAnalysis (ver. 3.4; Bruker Daltonik).

After MSI data acquisition, the slides were washed in 70% ethanol to remove the remaining matrix and then stained with hematoxylin and eosin (H&E). High-resolution digital images of the H&E stained tissues were then recorded using a Pannoramic MIDI slide scanner (3DHISTECH Ld., Budapest, Hungary) and co-registered to the MSI datasets in FlexImaging 3.0 (Bruker Daltonik).

### Histological Annotation and MSI Data Quality Control

The H&E stained images were histologically annotated by expert pathologists. To ensure only comparable regions of tissue were used in the subsequent statistical analysis, tumor regions were classified according to their degree of differentiation (well-, moderately-, or undifferentiated). These annotated regions-of-interest (ROIs) were then used to extract the tumor-specific metabolic signatures from the MSI data.

The quality of the MSI data from each ROI was then evaluated in ClinProTools 3.0 (Bruker Daltonik) by using the mass spectral preprocessing settings as reported in Supplementary Table 1: any dataset in which ≥40% of the spectra were excluded by ClinProTools’ built-in quality control metrics (either being non-alignable or a null spectrum) was disqualified for the further analysis. Note: owing to the strong presence of background peaks in the lower mass region, all ions up to *m/z* range 100 were excluded from the analysis.

### MSI Data Processing

The spectra from the annotated tumor areas of datasets that passed all quality filters embracing sample selection based on tumor cell fraction, MSI spectral quality assessment, and tissue integrity after H&E (Supplementary Table 2), were loaded into MATLAB R2013a (MathWorks, Natick, MA, USA) for further processing (all settings are summarized in Supplementary Table 3). There all spectra were normalized to their total-ion-count (TIC), and pixels with the 1% lowest and 1% highest TICs were discarded. Then a conservative peak picking using the LIMPIC package was performed on each sample’s average spectrum upon baseline removal (TopHat) and smoothing (Kaiser) [33]. Frequent peaks, which appear in over 98% of all samples, were used for recalibration of all spectra using the *msalign* routine (Bioinformatics toolbox) in MATLAB. After alignment, all tumor areas of the same differentiation degree were aggregated for each sample into basepeak mass spectra [34]. In a next step, all basepeak spectra were averaged across all samples to obtain the global basepeak mass spectrum in which metabolite peaks were detected by the *mspeaks* function (Bioinformatics toolbox; MATLAB) with a minimum relative basepeak intensity threshold of 0.4%. This final project-specific peak list was then used to extract the areas-under-the-curve (AUC) values from the average spectra of each patient’s tumor areas in two ways: first, to get the overall tumor profile for each patient, and second, to get the overall profile for each tumor differentiation degree for each patient.

### Statistical Association of Metabolic Signals to Survival of Patients

The association of MSI signals of all subsets with the follow-up data was investigated similarly as in our previous study of protein biomarkers of soft tissue sarcomas [3] in R (R Foundation for Statistical Computing, Vienna, Austria). Briefly, significant relationships between peak intensities and the overall/metastasis-free survival were first screened by using the Significance Analysis of Microarrays analysis in R (SAMR package). Metabolite ions with significant prognostic value (q ≤ 0.05; minimum median FDR) were then used to dichotomize patients based on the third quartile of intensity (data distribution and cutoff for one mass shown in Supplementary Figure 1). Differences in the overall/metastasis-free survival between the two resulting groups were compared by Kaplan-Meier curves and the *log rank* test using the *survival* package. *P*-values ≤0.05 were considered significant.

### Metabolite Identification Using High Mass Accuracy MALDI-FTICR

Additional tissue sections were prepared from patients whose MALDI-TOF-based MSI datasets contained the prognostic metabolite ions in high abundance (see above description). These tissue sections were then analyzed using an ultrahigh mass resolution mass spectrometer, namely a 9.4T MALDI Solarix XR Fourier transform ion cyclotron resonance mass spectrometer equipped with a dynamically harmonized ParaCell (Bruker Daltonik, Bremen, Germany). MSI was performed in negative mode using 500 laser shots per spot, laser frequency 1 kHz, and 100 μm pixel size. Data was acquired from *m/z* 50 to 1000 with a 512 k data point transient and an estimated resolution of 19,000 at *m/z* 400 Da. Data acquisition was performed using ftmsControl (Bruker Daltonik).

Metabolite identities were assigned on the basis of accurate mass and matched isotope distributions measured with a high field FTICR MS. The metabolites measured with the FTICR were assigned to peaks measured with the MALDI-TOF system on the basis of accurate mass, isotope patterns, and used matched spatial distributions as an additional constraint.

## Results and Discussion

Here we investigated whether MSI could identify metabolite biomarkers associated with survival and metastasis in high grade sarcomas. Prognostic metabolite signals were first identified based on MALDI-ToF-MSI data, then confirmed and identities assigned using high resolution, high mass accuracy MALDI-FTICR-MSI.

**Figure 1:**
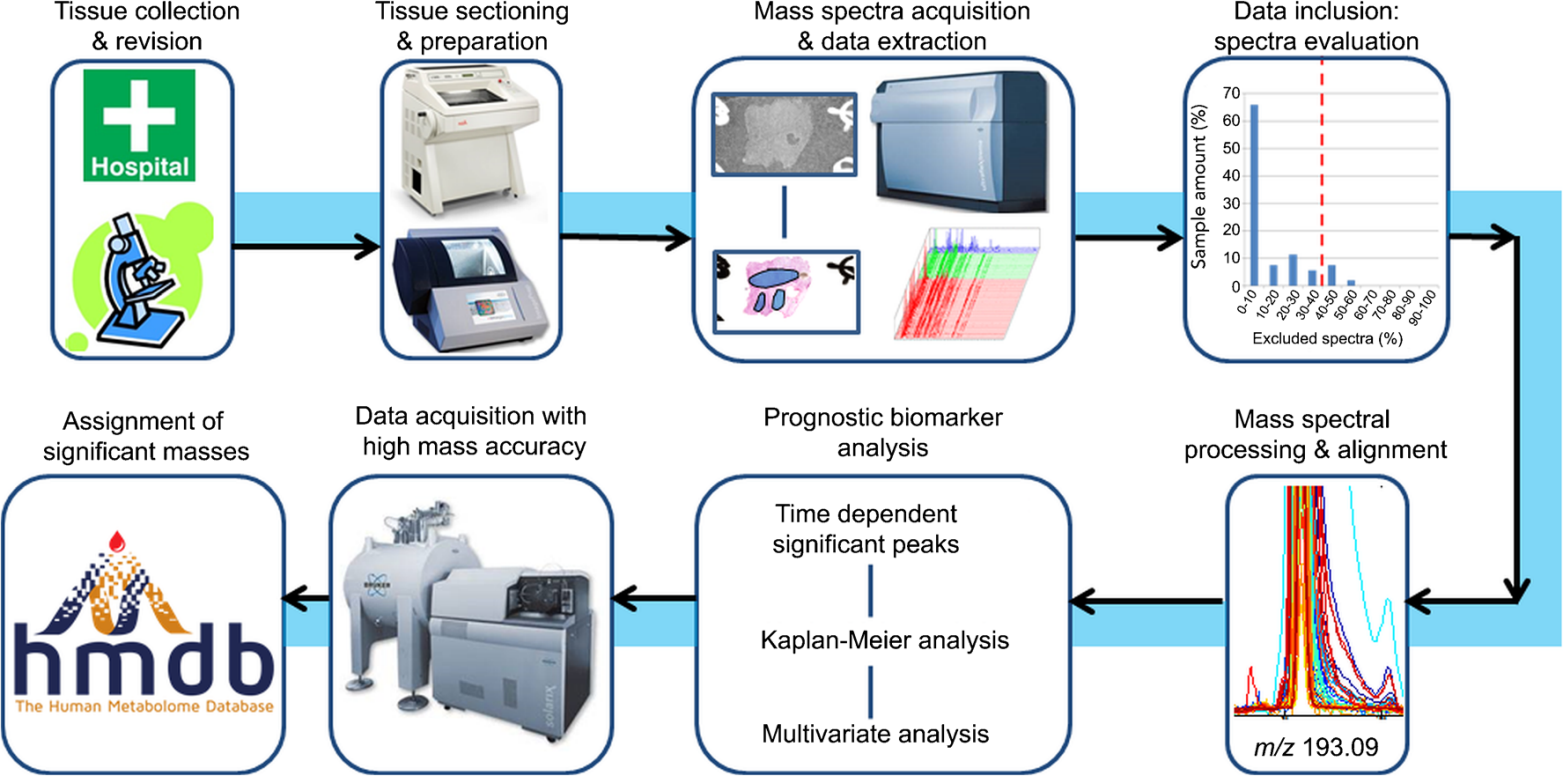
Study workflow. (1) Fresh frozen tissues with diagnosis of leiomyosarcoma, myxofibrosarcoma, undifferentiated pleomorphic sarcoma, and osteosarcoma were collected and revised by pathologists in LUMC. (2) Tissue samples were cryosectioned and the matrix 9-arminoarcridine applied. (3) MALDI-MSI datasets were acquired using a MALDI-TOF platform and spectra from regions of interest were extracted for analysis. (4) Datasets QC and inclusion criteria (full criteria listed in Supplementary Table 2). (5) Spectra from all the datasets were processed in MATLAB including sample alignment. (6) Masses that showed significant relationship between peak intensity and survival time were first selected using “significance analysis of microarrays,” then were analyzed using Kaplan-Meier survival analysis, and finally were evaluated to ensure biomarkers were independently correlated with the survival time. (7) Representative poor and good survival patient samples were measured using a MALDI-FTICR platform for high accuracy. (8) Significant masses with high accuracy were assigned based on human metabolome database and using isotope patterns as an additional constraint

High grade sarcomas can be highly heterogeneous histologically, with a single tumor containing areas of different histologic grade, necrotic regions, and a variable inflammatory cell infiltrate. This heterogeneity can introduce high measurement variance, complicating the search for molecular biomarkers [3]. Therefore, a histopathology-defined data analysis approach was utilized in which each tissue’s histology was first annotated by pathologists who specialized in soft tissue sarcomas. Each tissue section was first analyzed by MALDI MSI and then H&E stained. High resolution histologic images were recorded using a digital slide scanner, which were then registered to the MSI datasets using fiducial markers in FlexImaging. A virtual microdissection was then performed to isolate the mass spectra from designated tumor regions of interest. In this manner, the spectra from areas with comparable histologic grade (well differentiated, moderately differentiated, undifferentiated), free of inflammatory cell infiltrate, free of necrosis, and with high tumor cell content were extracted from each patient tissue. This cell-specific data was used for the statistical analysis. The workflow is depicted in Figure 1.

To control false positive identification of biomarkers, we used three sequential filters:

1. A Significance Analysis of Microarrays (SAM) [35] was used to screen for survival-associated peaks. SAM controls the false positive identification rate and was set here to 5%.
2. A log rank test on the dichotomized patient survival data was used to confirm the prognostic capability of the metabolite peaks.
3. Metabolite biomarker ions identified only detected in small groups of patients, less than 10, were omitted.

The clinical covariates (Table 1) did not exhibit any significant association to the patient survival data (age, gender, tumor type, adjuvant therapy; in all case the log-rank test resulted with *P* > 0.05), or the clinical covariate was significant but defined groups of too small sample size (e.g., only two neoadjuvantly treated patients) that a reliable statistical analysis could not be guaranteed. Similarly, the prognostic biomarkers were not significantly associated with the clinical covariates.

**Figure 2:**
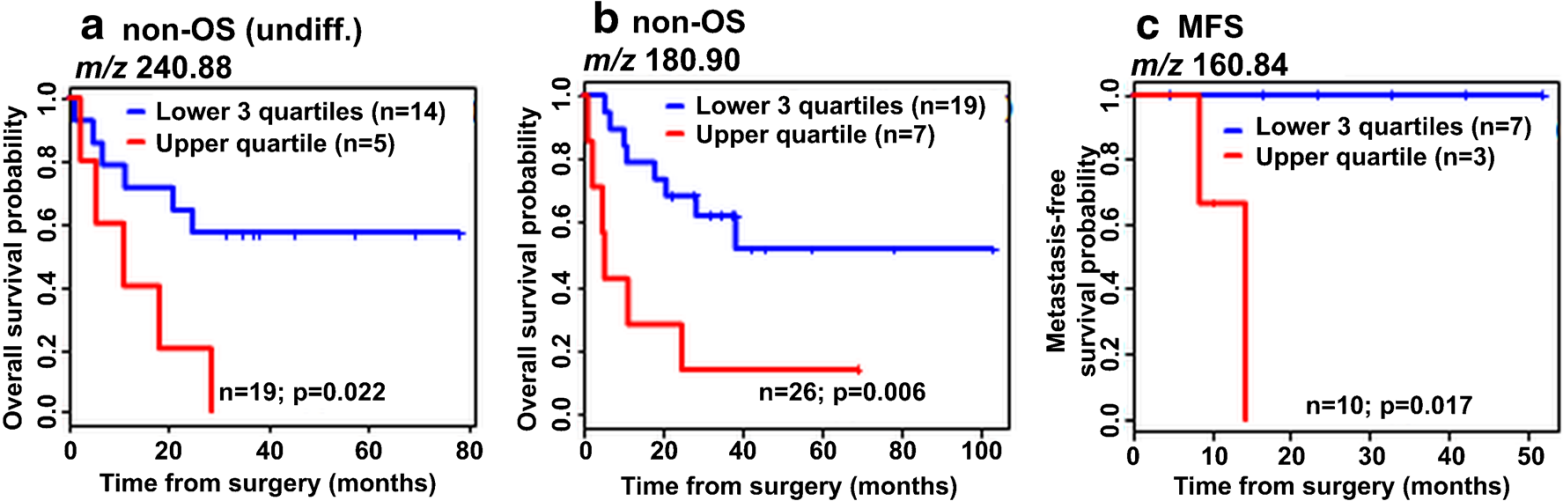
Kaplan-Meier survival plots of prognostic biomarkers discovered from MALDI-TOF MSI datasets. Prognostic metabolite ions were found from a sub-dataset of myxofibrosarcomas and from a dataset consisting of all soft tissue sarcomas (non-OS subset). **(a)** Shows a metabolite ion (*m/z* 240.88) that was found when the analysis was limited to undifferentiated areas, and was indicative for poor survival of soft tissue sarcoma patients; **(b)** and **(c)** show two metabolite ions (*m/z* 180.90 and *m/z* 160.84) indicating poor survival in soft tissue sarcoma patients and myxofibrosarcoma patients, respectively. *P*-values listed are calculated using a log-rank test in Kaplan-Meier analysis

The analysis revealed three metabolite ions that were significantly associated to patient survival in sarcoma patients: *m/z* 180.90 (*P* = 0.006) and *m/z* 240.88 (*P* = 0.022), both indicative of a poor overall survival in soft tissue sarcoma patients; and *m/z* 160.81 (*P* = 0.017) indicative of a poor metastasis-free survival in myxofibrosarcoma patients. The Kaplan-Meier plots are shown in Figure 2. In order to increase the sample size for the subgroups, leiomyosarcoma, myxofibrosarcoma, and undifferentiated pleomorphic sarcoma were grouped together as a single non-osteosarcoma (non-OS) group because these three subtypes are all soft tissue sarcomas, whereas osteosarcoma is primarily located in bone. Common biomarkers are beneficial from both a clinical and logistical viewpoint: for rare and sometimes diagnostically challenging tumors such as high grade sarcomas, which only present 1% of all malignancies and have over 50 histologic subtypes, a prognostic biomarker common to many/all subtypes would be more broadly applicable, even for cases in which a definitive diagnosis is uncertain.

**Figure 3:**
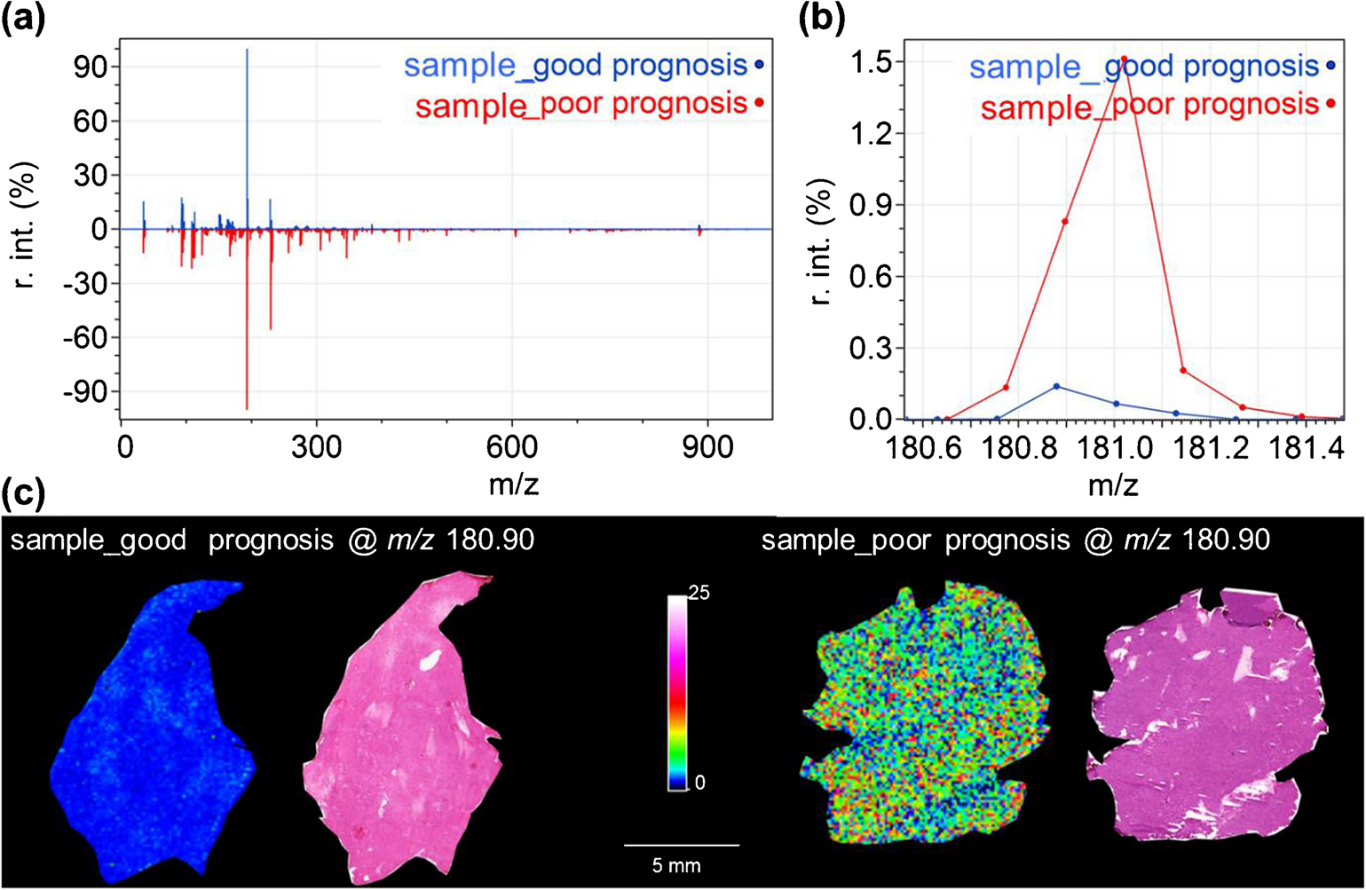
MALDI-TOF MSI data of the prognostic metabolite ion at *m/z* 180.90. **(a)** Shows a comparison of the average spectra of two soft tissue sarcoma samples, one with good prognosis (blue line) and one with poor prognosis (red line); **(b)** shows the magnification of *m/z* 180.90. Histologic images and MSI visualizations for these two samples are shown in **(c)**, confirming the higher detected intensity throughout the poor survival patient sample

The prognostic value of the metabolite ions were confirmed by visualizing the metabolite MSI data in patient samples with good and poor survival. Figure 3 shows the differential detection level of the metabolite ion at *m/z* 180.90 in two non-OS patients with differential survival.

**Figure 4:**
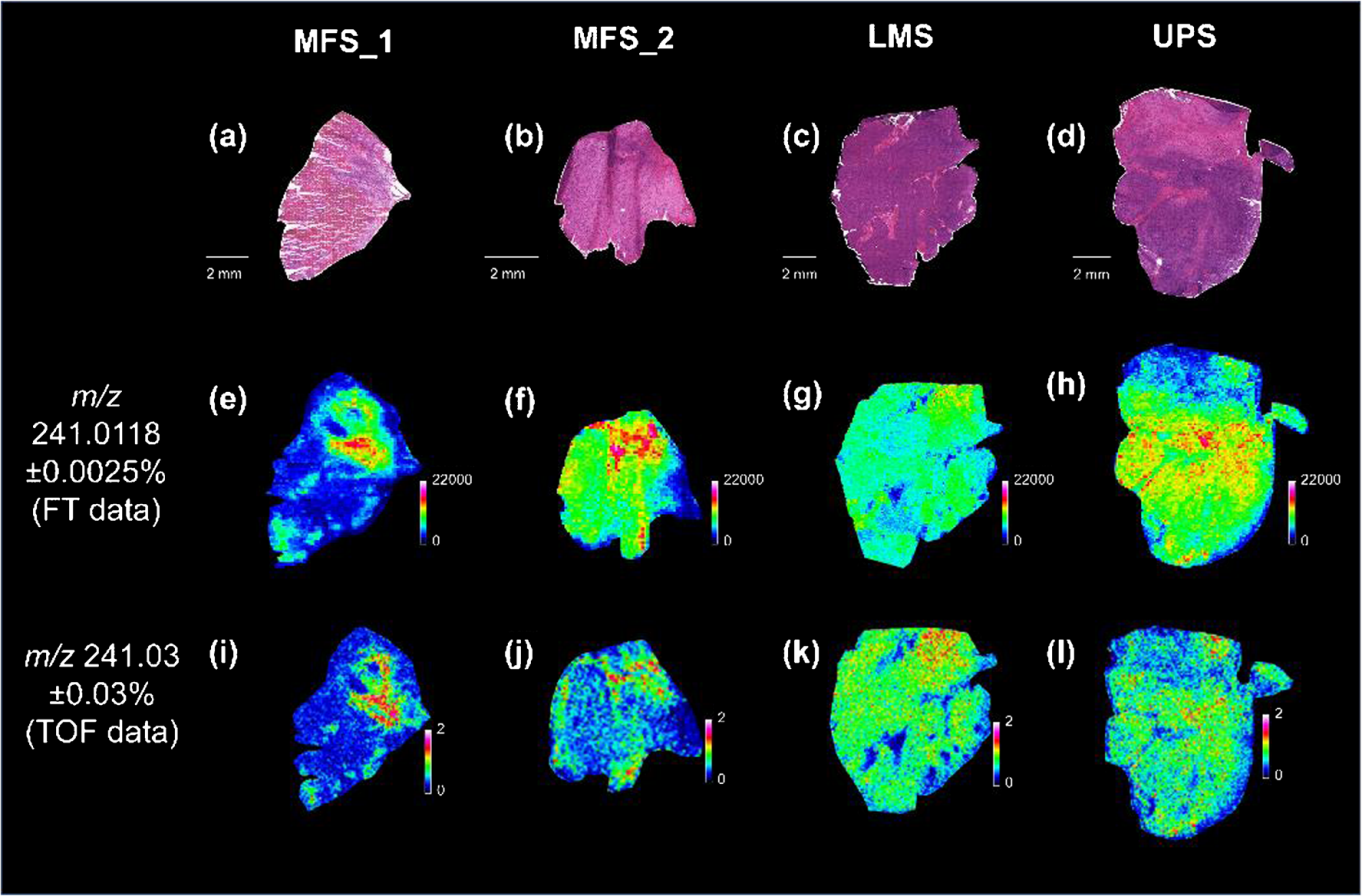
Comparison of MALDI MSI images recorded using the MALDI-TOF and MALDI-FTICR mass spectrometers. Histologic images of four samples are shown in **(a)**–**(d)**; **(e)**–**(h)** are the MS images of inositol cyclic phosphate obtained with the MALDI-FTICR, and **(i)**–**(l)** the MS images obtained with the MALDI-TOF instrument

The metabolite MALDI MSI datasets used for the statistical analysis were obtained using a MALDI-TOF mass spectrometer. The mass accuracy and mass resolution of the system does not allow for the unambiguous assignment of the prognostic metabolite ions. Several poor survival and good survival patient tissues were re-analyzed using a high field FTICR mass spectrometer, in order to obtain high mass resolution high mass accuracy MALDI MSI data. Despite significant differences in mass analyzer technology (the time scale of detection of the MALDI-TOF instrument is approximately 10^5^ times shorter than that of the FTICR) the mass spectral patterns were consistent, Supplementary Figure 4. As an example, the MS images from MALDI-TOF and MALDI-FTICR for *m/z* 241.0118 are shown in Figure 4. The mass accuracy of the FTICR experiments was obtained using the metabolites ATP, ADP, and AMP (images and experimental masses reported in Supplementary Figure 2), and was found to be less than 3 ppm (note: the MSI dataset was not aligned nor recalibrated). Accordingly, 3 ppm was the used for the maximum mass tolerance for querying metabolomics databases.

It is now well established that MALDI MSI, using a given matrix preparation, detects a consistent subset of metabolites. Metabolite identities were assigned on the basis of accurate mass, and matched isotope distributions measured with a high field FTICR system to previously reported metabolite ions. MS/MS was attempted but the low signal intensities of the metabolite peaks and the presence of isobaric ions (see Supplementary Figure 3) led to very noisy MS/MS spectra.

Within 2 ppm difference, the molecule at *m/z* 160.8417 was previously reported by Buck et al. at *m/z* 160.8420 in colon cancer and assigned as carnitine [27]. The molecule observed at *m/z* 241.0118 was assigned as inositol cyclic phosphate using the Human Metabolome Database with an error of 0.32 ppm and matching the isotope pattern (Supplementary Figure 3). The ion detected at *m/z* 180.9436 could not be assigned using the available databases and MALDI-MSI literature. Here we detected the abundant metabolites that were statistically associated to patient survival. Metabolites signals that could differentiate between the different histologic subtypes were also assessed, but none were statistically significant.

Carnitine is an amino acid derivate involved in lipid metabolism and is known to contribute to multiple diseases, including cancer [36]. Carnitine plays an essential role in transporting fatty acids to mitochondria with the help of carnitine palmitoyl transferase I (CPTI) for energy production by fatty acid oxidation (FAO) [37]. FAO and CPTI have been reported as emerging therapeutic targets in cancer [38]. Inositol (1,2-)cyclic phosphate belongs to the class of organic compounds known as cyclitols. It can be transformed to inositol 1-phosphate (IP), which is involved in inositol 1,4,5-trisphosphate and calcium (IP3/Ca2+) signaling system [39]. Altered expression of inositol IP3 receptor has been reported in different types of cancer and implicated in patient prognosis [40, 41].

The prognostic metabolite biomarker inositol cyclic phosphate was found to be statistically significant only when the analysis was focused on the undifferentiated regions of soft tissue sarcoma samples. Undifferentiated tumor cells are abnormal-looking cells compared with moderately differentiated or well differentiated tumor cells, and are considered to be more malignant. The increased statistical significance observed when the analysis was focused on undifferentiated tumor areas is consistent with molecular and/or measurement variance being introduced by histologic heterogeneity; the increased variance in the measured data undermines the ability to obtain statistically significant results.

The work reported here could be expanded in several different directions: (1) validate the results in a larger independent series; (2) assess whether inositol (1,2-)cyclic phosphate and carnitine are also prognostic for additional subtypes of soft-tissue sarcoma; (3) investigate the biological foundations of their prognostic value, which may ultimately lead to novel treatment strategies [42].

Sample degradation can be a very important issue in metabolomics research, and several methods have been investigated for maintaining metabolic integrity of tissue samples for MALDI MSI [43, 44]. If the purpose of the study is to investigate metabolic processes, metabolic integrity is key. Maintaining metabolic integrity is a major challenge for clinical tissue samples, as those obtained via resection (the majority of those that are available) will have undergone degradation during surgery as blood vessels are sealed, especially considering it has previously been shown that post-mortem degradation occurs more rapidly at body temperature than at room temperature [45]. The more limited scope of a biomarker study, in which the goal is to find mass spectral features that differentiate between patient groups, in this instance between poor and good survival, does not require maintenance of the system’s original metabolic state, only that the mass spectral features consistently differentiate between the groups. This work and previous work using MALDI of FFPE tissues [27], and DESI based analyses [28, 29], have clearly demonstrated the utility of metabolic signatures for differentiating between patient groups. For clinical research in which metabolic integrity needs to be kept as close as possible to the original physiologic state flash frozen needle biopsies are recommended.

## Conclusions

A prognostic metabolite biomarker discovery platform was setup for histologically heterogeneous high-grade sarcomas using fresh frozen tumor tissues. Inositol cyclic phosphate and carnitine were found to be potential generic prognostic biomarkers in soft tissue sarcoma patients.

## Electronic supplementary material

Below is the link to the electronic supplementary material.

ESM 1 (DOCX 1484 kb)
