# Supplementary material for: Prognostic Metabolite Biomarkers for Soft Tissue Sarcomas Discovered by Mass Spectrometry Imaging
Source: J Am Soc Mass Spectrom. 2016 Nov 21;28(2):376–83. doi: 10.1007/s13361-016-1544-4 (PMC5227002; doi:10.1007/s13361-016-1544-4)
Supplement: Supplementary file 1 — (DOCX 1484 kb) [file 13361_2016_1544_MOESM1_ESM.docx]

**Supplementary Information**

**Prognostic Metabolite Biomarkers for Soft Tissue Sarcomas Discovered by Mass Spectrometry Imaging**

Sha Lou^1^, Benjamin Balluff^1,2^, Arjen H.G. Cleven^3^, Judith V.M.G. Bovée^3^, Liam A. McDonnell^1,3,4*^

**Authors’ affiliations**

^1^ Center for Proteomics and Metabolomics, Leiden University Medical Center, Leiden, The Netherlands

^2^ Maastricht MultiModal Molecular Imaging institute M4I), Maastricht University, Maastricht, The Netherlands

^3^ Department of Pathology, Leiden University Medical Center, Leiden, The Netherlands

^4^ Fondazione Pisana per la Scienza ONLUS, Pisa, Italy

* Corresponding author

**Corresponding authors and reprint requests**:

Dr. Liam A. McDonnell

Center for Proteomics and Metabolomics

Leiden University Medical Center

Einthovenweg 20

2333 ZC Leiden

The Netherlands

E-mail: L.A.Mcdonnell@lumc.nl

Phone: +31 71 526 8744

Fax: +31 71 526 6907

**Table of contents**

Supplementary Figure 1. Data distribution of assigned mass………………………………3

Supplementary Figure 2. MALDI-FTICR-MSI data of AMP, ADP and ATP……………..4

Supplementary Figure 3. Isotope pattern confirmation of peak assignments………………5

Supplementary Figure 4. Comparison of MALDI-ToF and MALDI-FTICR spectra……...6

Supplementary Figure 5. Example MS images of with and without TIC normalization…..7

Supplementary Figure 6. Overview of metabolite ions detected by SAM with FDR <5%...8

Supplementary Table 1. ClinProTools setting for quality control………………………….9

Supplementary Table 2. Dataset inclusion criteria…………………………………………10

Supplementary Table 3. Parameters for mass spectral processing in MATLAB…………..11

Supplementary Figure 1. Data distribution of *m/z* 240.88.


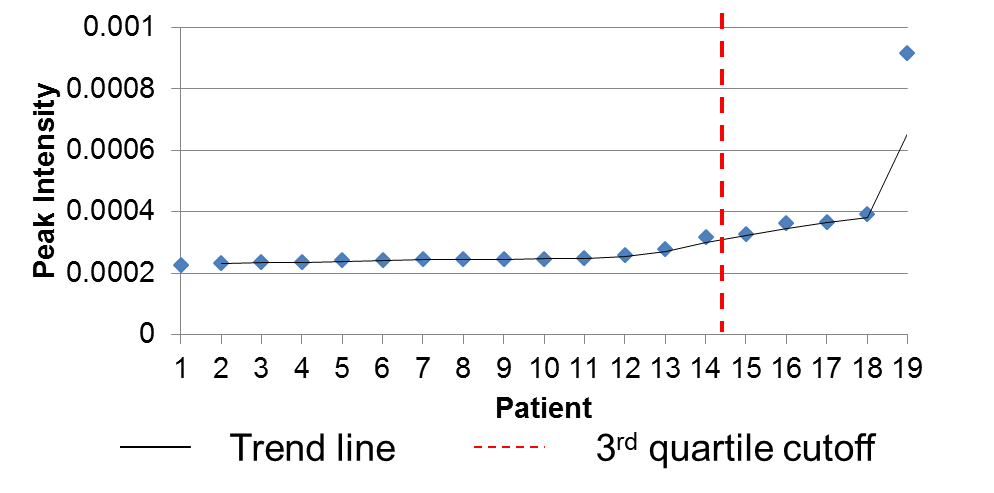


Supplementary Figure 2. MALDI-FTICR-MSI experimental data of AMP, ADP and ATP. Note datasets not recalibrated (improved mass accuracy could be obtained by recalibrating on AMP, ADP and ATP).


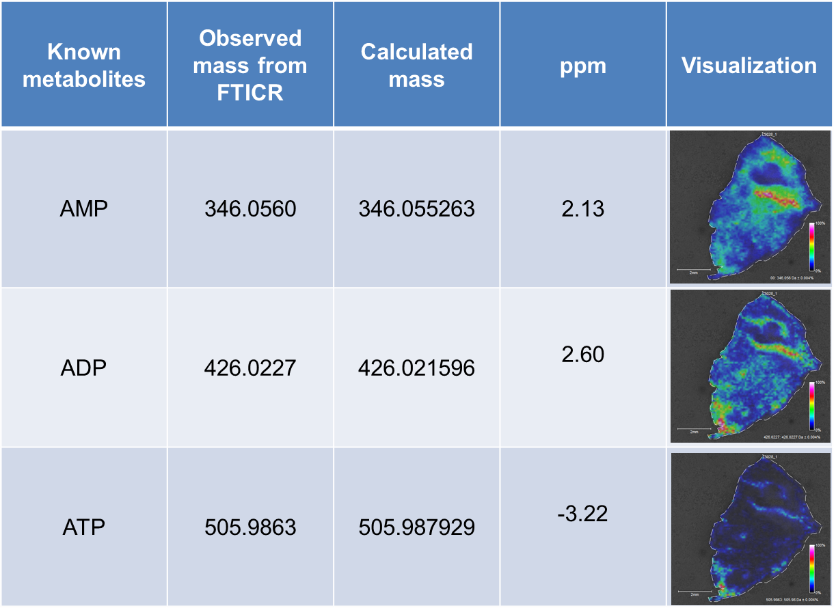


Supplementary Figure 3. Isotope pattern confirmation of peak assignments.


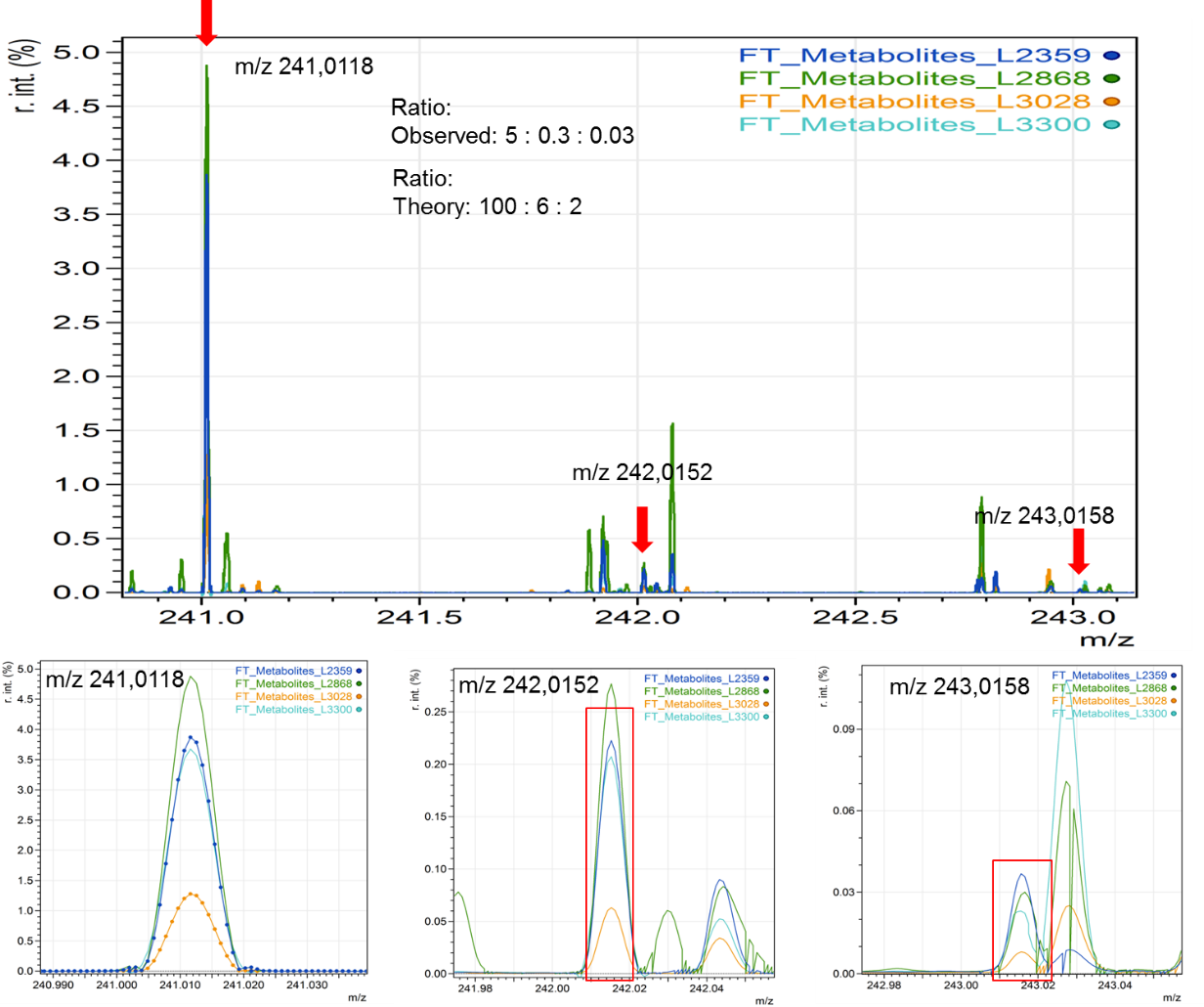


Supplementary Figure 4. Comparison of MALDI-ToF and MALDI-FTICR spectra obtained from sequential tissue sections. a) and b) show the total mass spectra obtained from the MALDI-ToF and MALDI-FTICR respecitively. c) – h) show close ups of the regions of the prognostic metabolites reported here (indicated with colored bars).

Supplementary Figure 5. Example MS images with and without TIC normalization. (a-c)/(g-i) are TIC normalized MS images (indicated with a T); (d-f)/(j-l) are the original images prior to TIC normalization, and which showed similar visualizations. The images are of the prognostic metabolite ions reported here, namely *m/z* 241.03, *m/z* 180.94 and *m/z* 160.87.

Supplementary Figure 6. Overview of the five metabolite ions detected by SAM analysis with FDR <5%. Upon Kaplan-Meier analysis two of the ions had P values greater than 0.05 (marked in red). Only those metabolite ions that were found to be associated with survival by SAM and Kaplan-Meier analysis were included in the main manuscript.


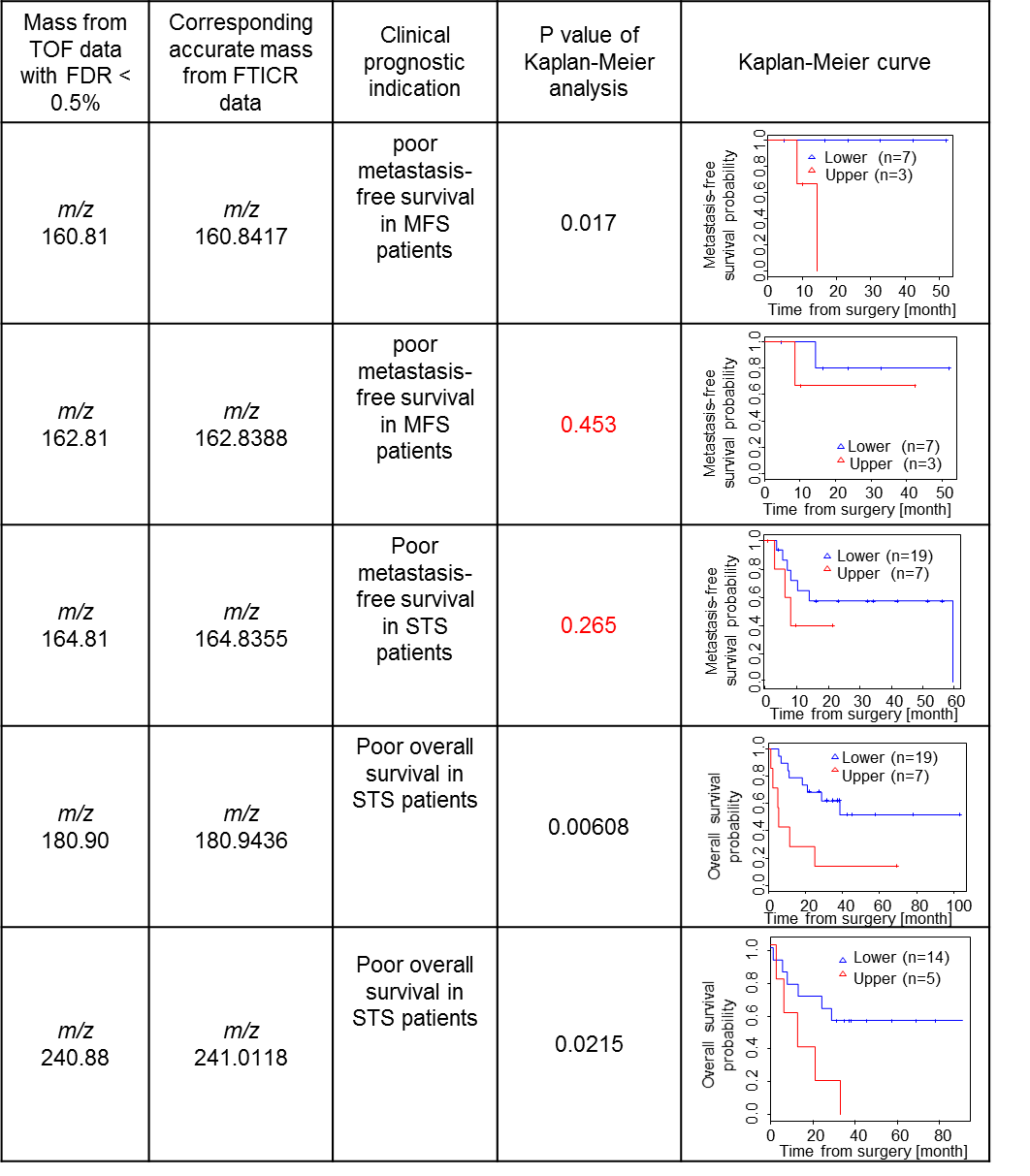


**Supplementary Table 1**. ClinProTools’ setting for quality control.

| **Parameter** | **Setting** |
| --- | --- |
| Resolution | 3000 |
| Baseline Subtraction | Top Hat Baseline |
|  | 10% Minimal Baseline Width |
| Mass Range | 100-1000 |
| Null spectra Exclusion | Enable |
| Recalibration | 1000ppm Maximal Peak Shift |
|  | 10% Match to Calibration Peaks |
|  | Exclude not Recalibratable Spectra |

**Supplementary Table 2**. Datasets inclusion criteria.

| Sample selection criteria | Viable area | 60% |
| --- | --- | --- |
|  | Sample source | Primary tumors |
|  | Consistent diagnosis | Yes |
| MSI quality control | Excluded spectra %) | <40% |
|  | Measurement bias | Randomized measurement sequence |
| Histology evaluation | The integrity of the tissue section | >= 70% ^1^ |

1) several sections (mainly bone sarcoma samples) did not survive the tissue wash to remove excess matrix or the histological staining procedure.

**Supplementary Table 3.** Parameters for spectra processing in MATLAB.

| ***Phase*** | ***Parameter*** | ***Value*** |
| --- | --- | --- |
| **Peak picking on sample spectra for alignment** | | |
|  | Spectrum to use | Mean TIC |
|  | Kaiser smoothing window [data points] | 25 |
|  | Resampling rate [Da] | 0.01 |
|  | M/z block [Da] | 20 |
|  | Baseline subtraction | TopHat with filter width 100,000 |
|  | Minimum signal-to-noise | 5 |
|  | Minimum half peak width | 0.03 |
|  | Number of reference peaks | 5 (equally weight) |
|  | Peak clustering tolerance [ppm] | 1500(*m/z* 0-200);  800(*m/z* 200-500);  600(*m/z* 500-1000) |
|  | Minimum peak detection rate | 98% |
| **Alignment** | | |
|  | Width of pulses msalign function) [Da] | 0.05 |
|  | Maximum shift [Da] | [-0.3,0.3] |
|  | Resampling rate [Da] | 0.01 |
| **Peak picking on global mass spectrum** | | |
|  | Spectrum to use | Basepeak spectrum |
|  | Smoothing window [data points] | 15 (lowess) |
|  | TopHat baseline subtraction width [data points] | 100000 |
|  | Minimum signal-to-noise | 0.4% of base peak |
| **Read-out of data and processing** | | |
|  | Use intensity or area of peaks | Area |
|  | Spectrum normalization | TIC |
|  | Remove extreme mass spectra according to TIC | 1% highest and 1% lowest |
|  | Offset for spatial arrangement of samples [px] | 10 |
